# Geographical and climatic distribution of lentil-nodulating rhizobia in Iran

**DOI:** 10.1093/femsec/fiae046

**Published:** 2024-04-08

**Authors:** Hossein Kari Dolatabad, Vahid Alah Jahandideh Mahjenabadi

**Affiliations:** Soil Biology and Biotechnology Department, Soil and Water Research Institute, Agriculture Research, Education and Extension Organization, Meshkin Dasht Road, Karaj 31785-311, Iran; Soil Biology and Biotechnology Department, Soil and Water Research Institute, Agriculture Research, Education and Extension Organization, Meshkin Dasht Road, Karaj 31785-311, Iran

**Keywords:** *Lens culinaris*, multilocus sequence analysis, phylogeny, *Rhizobium*, symbiotic genes

## Abstract

Lentil is one of the most important legumes cultivated in various provinces of Iran. However, there is limited information about the symbiotic rhizobia of lentils in this country. In this study, molecular identification of lentil-nodulating rhizobia was performed based on 16S–23S rRNA intergenic spacer (IGS) and *recA, atpD, glnII*, and *nodC* gene sequencing. Using PCR-RFLP analysis of 16S–23S rRNA IGS, a total of 116 rhizobia isolates were classified into 20 groups, leaving seven strains unclustered. Phylogenetic analysis of representative isolates revealed that the rhizobia strains belonged to *Rhizobium leguminosarum* and *Rhizobium laguerreae*, and the distribution of the species is partially related to geographical location. *Rhizobium leguminosarum* was the dominant species in North Khorasan and Zanjan, while *R. laguerreae* prevailed in Ardabil and East Azerbaijan. The distribution of the species was also influenced by agroecological climates; *R. leguminosarum* thrived in cold semiarid climates, whereas *R. laguerreae* adapted to humid continental climates. Both species exhibited equal dominance in the Mediterranean climate, characterized by warm, dry summers and mild, wet winters, in Lorestan and Kohgiluyeh-Boyer Ahmad provinces.

## Introduction

Lentil (*Lens culinaris*), belonging to the genus *Lens* of the *Viceae* tribe in the Leguminosae family, plays significant roles in the human diet and soil fertility maintenance. It has high protein and micronutrient contents (Abraham 2015, Ghanem et al. 2015) and improves soil fertility through the addition of nitrogen (N), carbon, and organic matter (Sarker and Erskine 2006, Abraham 2015). The world lentil production reached 5.77 million tons in 2021, with Canada being the major producer contributing up to 1.6 million tons (FAOSTAT 2021). Lentils are essential to the Iranian household diet, meeting the protein needs of the majority (Hashemzadeh and Monirifar 2016). Iran possesses significant potential as a lentil-producing country and ranks ninth globally in terms of cultivated area dedicated to lentil production. In 2021, lentil production in Iran amounted to 79 750 tons, contributing to a world share of 1.07% (FAOSTAT 2021). Due to the increasing population in Iran, the need to boost legume production, especially lentils, is greater than ever. Traditionally, this has been achieved through the use of chemical fertilizers to enhance production per unit area and expand the cultivation area of legumes. However, frequent droughts and land degradation resulting from excessive use of chemical fertilizers and intensive tillage operations have led to a decline in arable land area in Iran. Consequently, there is a pressing need to reevaluate methods for enhancing plant performance (Adesemoye et al. 2009, Abadi et al. 2020, 2021).

Lentils have the capability to establish symbiotic relationships with rhizobia, which can fix atmospheric N and meet the nutritional needs of legumes. This symbiosis has the potential to have environmental and agronomic benefits by reducing the utilization of chemical N fertilizers in agriculture (Riah et al. 2014). Integrating lentils into crop rotations with cereal crops promotes sustainable systems. The symbiotic relationship with rhizobacteria enhances the N content of the system, with reported levels of up to 107 kg ha ^−1^ (Abraham 2015). The arid and semiarid zones of Iran face significant limitations for crop production, including low rainfall, water deficits, poor soil fertility, and high soil salinity. These factors have adverse effects on symbiotic associations and N fixation. Therefore, the agronomic and ecological impacts of rhizobia partnerships depend on their symbiotic properties and their ability to adapt to environmental constraints (Riah et al. 2014). Similar to other legumes, lentils rely on effective rhizobia to fix atmospheric N. Accurate identification of symbiotic rhizobia of lentils is a crucial step in producing rhizobia inoculants. It is necessary to confirm the efficiency of rhizobia strains through greenhouse and field experiments, ultimately leading to increased lentil production (Anglade et al. 2015). 16S rRNA has low phylogenetic power at the species level, so accurate identification of closely related rhizobia strains usually requires phylogenetic analyses based on multilocus sequence analysis (MLSA), including different housekeeping genes or other techniques such as whole genome sequence analysis (Rashid et al. 2014, Aguilar et al. 2018, Sijilmassi et al. 2020).

Specificity is crucial for legume–rhizobia symbiotic associations; typically, a specific rhizobia species or biovar infects only a limited number of host plant species (Riah et al. 2014). The species *Rhizobium leguminosarum* currently comprises three symbiovars, each associated with specific host plants. These symbiovars include *phaseoli, trifolii*, and *viciae*, which mediate nodulation in the tribes of *Phaseolus, Trifolium*, and *Viciae*, respectively. Additionally, symbiovar *viciae* strains have been reported in *Rhizobium fabae* and *Rhizobium pisi* (Rashid et al. 2014). Lentils form a symbiotic relationship with *R. leguminosarum* biovar *viciae*, enabling atmospheric N fixation. However, reports on lentil symbiotic *Rhizobium* vary depending on geographical location and specific regional conditions.

Phylogenetic analyses of housekeeping genes (16S rRNA, *recA, atpD*, and *glnII*) and nodulation genes (*nodC, nodD*, and *nodA*) were conducted on 36 bacterial isolates to assess bacterial diversity and identify rhizobia nodulating lentil in Bangladesh. The majority of isolates were associated with *Rhizobium etli* and *R. leguminosarum*. Results of phylogenetic analyses indicated that the nodulation genes are linked to *R. leguminosarum* biovar *viciae* but form a distinct cluster. MLSA, DNA fingerprinting, and phenotypic characterizations suggested the involvement of at least three clades in lentil nodulation. These clades differed from the *R. etli*–*R. leguminosarum* groups (Rashid et al. 2014) and were subsequently identified as three new species: *Rhizobium bangladeshense, Rhizobium lentis*, and *Rhizobium binae*. Taha et al. (2018) conducted a study based on the molecular phylogeny of housekeeping genes from isolates gathered from 40 cultivated fields in the primary lentil production regions of Morocco. Their findings demonstrated that *Rhizobium laguerreae* serves as the primary symbiont of cultivated lentils in Morocco. The phylogenetic reconstruction of 26 strains nodulating lentils in Ethiopia, based on *recA, atpD*, and *glnII* genes, revealed three distinct sublineages (Clades I–III). Genospecies I and II were identified as *R. etli* and *R. leguminosarum*, respectively. However, Genospecies III was suggested to potentially represent an unnamed *Rhizobium* species (Tena et al. 2017). Young et al. (2021) constructed a phylogeny based on concatenated sequences of 120 universal genes. They concluded that the *R. leguminosarum* species complex comprises 18 distinct genospecies, along with seven unique strains that are not placed in these genospecies. Among these genospecies, five include the type strains of named species: *R. laguerreae, R. sophorae, R. ruizarguesonis, R. indicum*, and *R. leguminosarum*.

No studies have yet investigated the genetic diversity and taxonomic status of lentil rhizobia in Iran using the MLSA approach. Previous studies have mainly focused on the impact of symbiosis on lentil growth. Therefore, the objectives of this study were: (i) to explore the genetic diversity and population structure of rhizobia nodulating lentils adapted to the environmental conditions of Iran; and (ii) to characterize lentil rhizobial isolates through sequencing analysis of 16S rRNA, protein-encoding housekeeping genes (*recA, atpD*, and *glnII*), and the nodulation gene (*nodC*).

## Materials and methods

### Collection of nodules and rhizobia isolation

**Figure 1.**
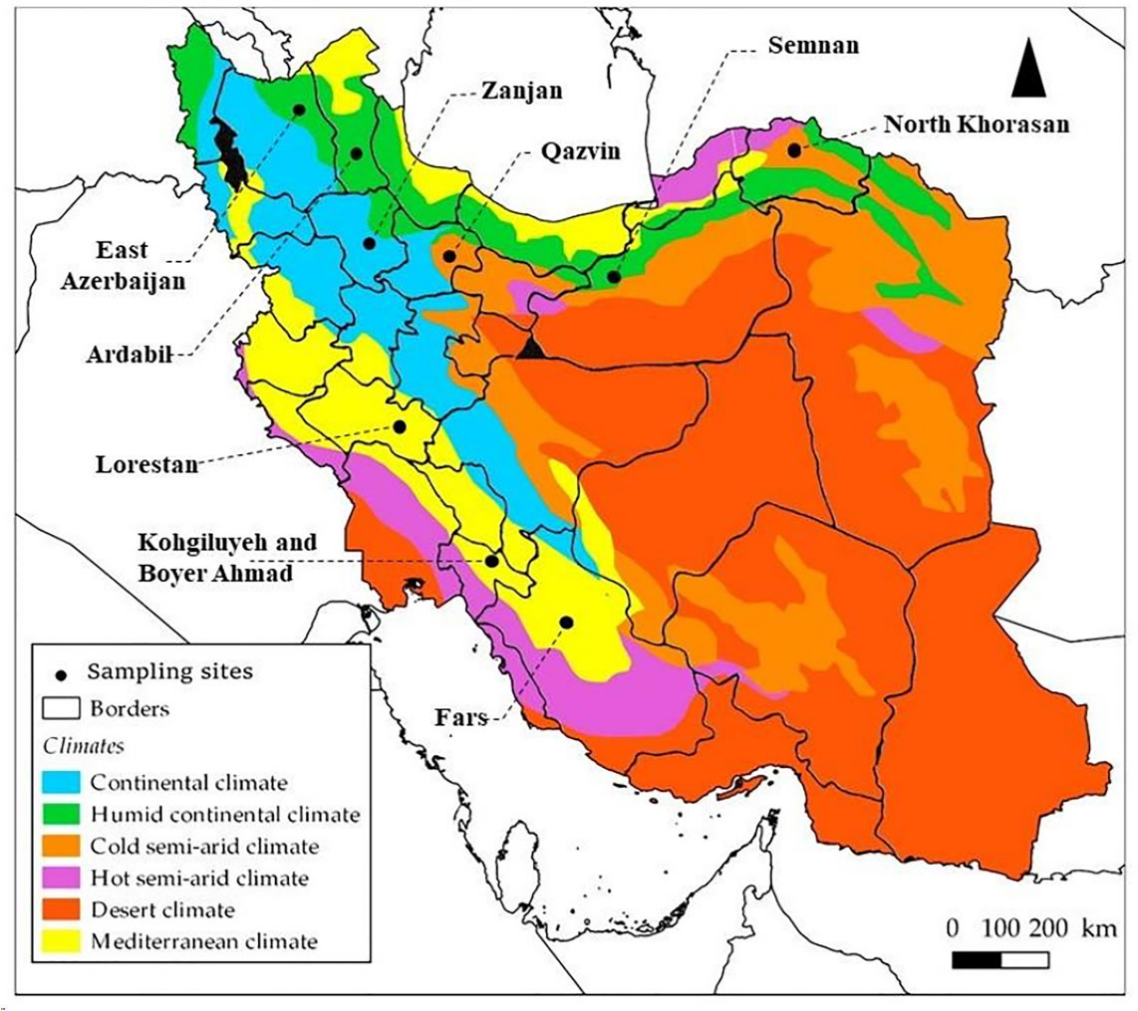
Map of Iran showing sampling sites and climatic regions.

A total of 80 different lentil farms located in the nine principal provinces of lentil production in Iran (Fars, Lorestan, Kohgiluyeh-Boyer Ahmad, Qazvin, Ardabil, North Khorasan, Zanjan, East Azerbaijan, and Semnan) were randomly selected. Sampling sites were distributed across humid continental, Mediterranean, cold semiarid, and continental climates (Fig. 1).

Nodulated roots and soil samples were collected from lentil farms during the flowering stage from April to May 2018. These farms have a history of lentil cultivation and have never been artificially inoculated with rhizobia. The nodulated roots were washed with sterile distilled water, dried with tissue paper, and then preserved on silica gel until further experiments. Soil samples were utilized for the rhizobia-trapping experiment. Therefore, the lentil rhizobia collection comprised bacteria isolated from nodules collected in the farms and nodules obtained via bacterial trapping in the greenhouse.

To trap rhizobia from the soil, lentil seeds (Bilesavar) underwent surface sterilization by immersion in 96% ethanol for 30 s and then in 0.1% mercury chloride (HgCl_2_) for 2 min, followed by rinsing 10 times with sterile distilled water. Subsequently, the seeds were transferred to agar plates for germination and kept at room temperature for 48 h. Disinfected plastic pots were filled with various soil samples, and five seedlings were planted in each pot, with three pots allocated for each soil sample. The plants were cultivated in the greenhouse under controlled conditions at 25°C under 16 h light/8 h dark cycles for a duration of 2 months. The plants were uprooted, and the roots were rinsed with sterile distilled water. Nodules were separated from the roots obtained from both the field and the greenhouse culture. The nodules were sterilized by immersing them in 96% alcohol for 30 s and then in 30% sodium hypochlorite for 2 min, followed by rinsing ten times with sterile distilled water. For the isolation of rhizobia, a single nodule was crushed in sterile distilled water using a homogenizer under aseptic conditions. The extract was streaked onto yeast-extract mannitol agar (YEMA) plates containing Congo red (Vincent 1970) and incubated at 28°C for 3–5 days. The isolates were purified by streaking several times on YEMA plates, and then single colonies were maintained on agar slants at 4°C until subsequent experiments.

### Plant infection assay

To confirm the ability of isolates to form effective nodules on lentils, plant infection assays were conducted. Lentil seeds were surface-sterilized and germinated as previously described. The seedlings were then transferred to holes created in test tubes (300 mm × 35 mm) filled with Fahraeus agar medium containing 1% (w/v) CaCO_3_. A 1 ml rhizobial suspension (~10^8^ cells ml^−1^) was used to inoculate 4-day-old seedlings. The tubes were placed in a growth chamber at 25°C under 16 h light/8 h dark cycles for 4 weeks. During this period, sterile distilled water and Jensen’s N-free solution were used for plant irrigation. For each isolate, nodulation tests were conducted in three replicates, while negative control plants were left uninoculated. Finally, nodule development was evaluated.

### RFLP analysis of the intergenic spacer gene

Based on the plant infection assay, 116 isolates were obtained, and DNA extraction was performed using the genomic DNA extraction kit (CinnaGen Co., Ltd. Tehran, Iran). The genomic region intergenic spacer (IGS) between the 16S and 23S rRNAs was amplified using primers FGPS1490 and FGPL1320, with the PCR conditions provided in Table 1. The enzymes BshFI and MspI were used separately to digest the IGS fragments at the GG“CC and C”CGG sites, respectively. Subsequently, the restriction fragments were separated by electrophoresis in a 2% (w/v) agarose gel containing DNA Safe Stain. The IGS restriction patterns were stained, visualized under UV light, and analyzed using Bio-Vision software. The unweighted pair group method with arithmetic averages (UPGMA) (Nei and Li 1979) was utilized for cluster analysis through the NTSYS 2.1 program (Rohlf 1998). Additionally, the distribution of *Rhizobium* populations was statistically compared among and within provinces and four climatic zones by analysis of molecular variance (AMOVA) using GenAlex software version 6.5 (Peakall and Smouse 2012).

**Table 1.** Primer sequences and PCR conditions used in this study.

Target gene	Primer	Sequence (5′–3′)	PCR conditions	Amplified Length (bp)	Aligned sequences (bp)	Reference
**IGS**	FGPS1490 FGPL1320	TGCGGCTGGATCACCTCCTT CCGGGTTTCCCCATTCGG	3 min 94°C, 35 × (1 min 94°C, 1 min 55°C, 2 min 72°C), 7 min 72°C	1400	–	Laguerre et al. (1996)
**16S rRNA**	27F 1492R	AGAGTTTGATCCTGGTCAGAACGCT CGGTTACCTTGTTACGACTT	5 min 94°C, 30 × (45 s 94°C, 1 min 52° C in 2 min 72°C), 7 min 72°C	1450	1326	Jiang et al. (2006)
***nodC***	nodCF nodCI	AYGTHGTYGAYGACGGTTC CGYGACAGCCANTCKCTATTG	5 min 95°C, 35 × (1 min 94°C, 45 s 55°C, 1.5 min 72°C), 7 min 72°C	1000	884	Laguerre et al. (2001)
***glnII***	TsglnIIf TsglnIIr	AAGCTCGAGTACATCTGGCTCGACGG SGAGCCGTTCCAGTCGGTGTCG	5 min 95°C, 35 × (45 s 95°C, 30 s 58°C, 1.5 min 72°C), 7 min 72°C	700	648	Stepkowski et al. (2005)
***recA***	63F 504R	ATCGAGCGGTCGTTCGGCAAGGG TTGCGCAGCGCCTGGCTCAT	5 min 95°C, 35 × (1 min 94°C, 1 min 65°C, 1 min 72°C), 7 min 72°C	500	478	Gaunt et al. (2001)
***atpD***	273F 771R	SCTGGGSCGYATCMTGAACGT GCCGACACTTCCGAACCNGCCTG	5 min 95°C, 35 × (1 min 94°C, 45 s 55°C, 1 min 72°C), 7 min 72°C	550	518	Gaunt et al. (2001)

### Amplification and sequencing of genes

The 16S rRNA gene, along with three chromosomal housekeeping genes (*recA, atpD*, and *glnII*), and the symbiosis-related gene (*nodC*) of 27 representative strains from the IGS-RFLP groups were amplified. PCR conditions and primer sequences used for gene amplification are provided in Table 1. For sequencing, PCR products were purified using a PCR purification kit (CinnaGen Co., Ltd.) and sequenced by Bioneer Co., South Korea. A total of 135 sequences were generated and deposited in GenBank under the accession numbers: *recA* (ON454898–ON454924), *glnII* (ON478264–ON478290), *atpD* (ON454925–ON454951), *nodC* (ON454952–ON454978), and 16S rRNA (ON428638–ON428664). The Rhizobial strains were deposited in the Culture Collection of Soil Microorganisms (CCSM) at the Soil and Water Research Institute (SWRI), Iran.

### Phylogeny and nucleotide polymorphisms analyses

The sequences obtained were checked and assembled with the Vector NTI Advance^TM^ 10 software. Subsequently, each gene sequence was compared with the corresponding genes of reference *Rhizobium* species in the NCBI database using nucleotide BLAST. Multiple sequence alignments for all isolates and reference *Rhizobium* species (Table 2) were conducted using the CLUSTAL W program from MEGA version 11. Phylogenetic trees were constructed using the maximum likelihood method with the Kimura 2-parameter model in MEGA version 11 (Tamura et al. 2021). The robustness of the tree topology was assessed through bootstrap analysis with 1000 replications of each sequence using MEGA 11 software. The phylogenetic tree of concatenated genes was generated from the sequence alignments of all isolates and the described reference species. Nucleotide polymorphisms, such as nucleotide diversity, the number of haplotypes, and haplotype diversity, were calculated using DNaSP V6 software (Rozas et al. 2017).

**Table 2.** Reference/type strains used in this study.

Species	Type strain	GenBank accession number (recA/glnII/atpD)	Host Plant	Geographical origin
*R. bangladeshense*	BLR175^T^	JABDXG000000000	*L. culinaris*	Bangladesh
*R. etli*	CFN 42^T^	CP000133	*Phaseolus vulgaris*	Mexico
*R. fabae*	CCBAU 33202^T^	RJJU00000000	*Vice faba*	China
*R. indicum*	JKLM 12A2^T^	CP054021	*Pisum sativum*	India
*R. laguerreae*	WSM1455	CP088090	*Vice faba*	Greece
*R. laguerreae*	FB206^T^	MRDM00000000	*Phaseolus vulgaris*	Tunisia
*R. leguminosarum* bv*. viciae*	USDA 2370^T^	MRDL00000000	*Pisum sativum*	USA
*R. leguminosarum* bv*. viciae*	248	CP048280	*Vicia faba*	United Kingdom
*R. leguminosarum*	GLR17	CP071626	*L. culinaris*	Germany
*R. lentis*	BLR27^T^	JABDYH000000000	*L. culinaris*	Bangladesh
*Rhizobium mongolense*	USDA 1844^T^	VISO00000000	*Medicago ruthenica*	China
*R. phaseoli*	ATCC 14482^T^	JAQJCF000000000	*Phaseolus vulgaris*	Brazil
*R. pisi*	DSM 30132^T^	RJJT00000000	*Pisum sativum*	Unknown
*R. ruizarguesonis*	UPM1133^T^	PQIG00000000	*Pisum sativum*	Italy
*R. sophorae*	CCBAU 03386^T^	JABFCN000000000	*Sophora flavescens*	China
*Rhizobium vallis*	CCBAU 65647^T^	RJTH00000000	*Phaseolus vulgaris*	China
*Rhizobium aegyptiacum*	1010^T^	KU664569/-/ KU664561	*Trifolium alexandrinum*	Egypt
*Rhizobium aegyptiacum*	950	KF483575/KF483573/KF483571	*Trifolium alexandrinum*	Egypt
*R. leguminosarum* bv. *viciae*	CCBAU 65264	GQ323679/GQ323642/GQ323603	*Vicia faba*	China
*R. leguminosarum* bv*. viciae*	GLR19	KC679463/KC679575/KC679521	*L. culinaris*	Bangladesh
*R. leguminosarum* bv*. viciae*	CCBAU 11117	GQ323667/GQ323630/GQ323591	*Vicia faba*	China
*R. laguerreae*	FB14022	JN558690/JN558680/JN558670	*Vicia faba*	Tunisia
*R. laguerreae*	FB310	JN558682/JN558672/JN558662	*Vicia faba*	Tunisia

### Average nucleotide identity

Average nucleotide identity (ANI) analysis was performed using the ANI calculator provided by ChunLab (Seoul, Korea) (http://www.ezbiocloud.net/tools/ani) and Kostas Lab (http://enveomics.ce.gatech.edu/ani). ANI was determined between strains of this study and closely related type species of the genus *Rhizobium*.

## Results

### Bacterial isolation

In this study, a total of 218 strains were isolated from various provinces of Iran (Table 3). The number of rhizobial isolates obtained from each province varied, primarily due to differences in the number of soil samples collected. For instance, Ardabil province, which boasts the largest area of lentil cultivation in Iran, had a greater number of soil samples collected, consequently resulting in a higher number of rhizobial isolates obtained from this region. In terms of climate, Ardabil, East Azerbaijan, and Semnan provinces, characterized by a humid continental climate, yielded 67 isolates. On the other hand, Lorestan, Kohgiluyeh-Boyer Ahmad, and Fars provinces, featuring a Mediterranean climate with warm, dry summers and mild, wet winters, yielded 74 isolates.

**Table 3.** The number of isolated rhizobia in each province.

Provinces	Number of isolates
North Khorasan	34
Fars	13
Ardabil	46
Kohgiluyeh-Boyer Ahmad	34
Lorestan	27
Ghazvin	15
Zanjan	28
East Azerbaijan	12
Semnan	9

Based on the plant infection assay, 116 isolates were capable of forming nodules and were subsequently selected for amplification of the 16S–23S rRNA IGS region. The highest percentage of rhizobial isolates obtained based on the plant infection assay was 78% from Ardabil province, followed by Lorestan and Kohgiluyeh-Boyer Ahmad provinces, with nodulation rates of 59% and 56%, respectively. In North Khorasan, Zanjan, and Kohgiluyeh-Boyer Ahmad provinces, the predominant species identified was *R. leguminosarum*, accounting for 83%, 79%, and 53% of isolates, respectively. Conversely, in Ardabil and Lorestan provinces, *R. laguerreae* was identified as the dominant species, constituting 61% and 56% of isolates, respectively. The geographical origin and IGS-RFLP groups of the isolates are listed in Table S1 (Supporting Information).

### PCR-RFLP of 16S–23S rRNA IGS region

**Figure 2.**
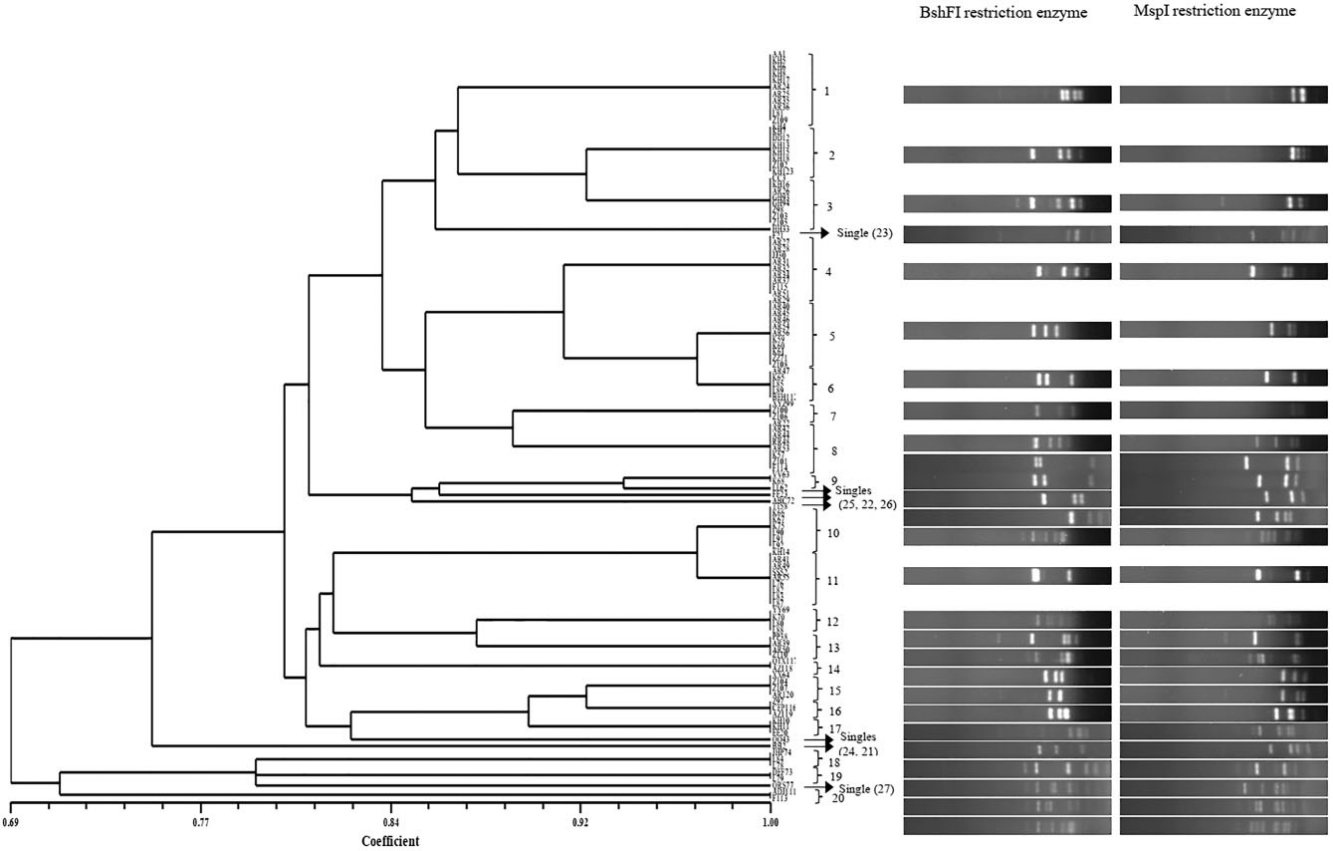
Genetic relationships among the rhizobia isolated from *L. culinaris* by the IGS PCR–RFLP analysis. The dendrogram was constructed using the UPGMA method.

The amplified DNA products ranged in size from 1350 to 1400 bp. RFLP analysis of isolates revealed that BshFI produced two to nine bands ranging in size from 100 to 520 bp, while MspI generated three to nine bands ranging from 100 to 530 bp (Fig. 2). Based on the combined patterns obtained from the RFLP results, the 116 isolates were classified into 20 groups and seven unclustered strains (Fig. 2; Table S1, Supporting Information). Therefore, one representative was selected from each group, and the subsequent steps were carried out with 27 selected isolates.

Four climatic zones (humid continental, Mediterranean, cold semiarid, and continental climates) were analyzed by AMOVA analysis to determine genetic variation among and within populations (Table 4). An overall significant differentiation was observed among the four climatic zones, with the variation among populations accounting for 12% of the total variation. With the exception of Semnan province, which had only one rhizobial isolate and therefore was not included in the analysis, the remaining provinces underwent AMOVA analysis. The results showed that 86% of the variation was within provinces, while 14% was among provinces. Notably, no significant differentiation among provinces in the Mediterranean climate and cold semiarid climate was observed. However, differentiation among provinces in the humid continental climate was found to be significant (Table 4).

**Table 4.** Genetic differentiation of the rhizobial populations according to their geographical origins and climates by AMOVA based on PCR-RFLP of 16S–23S rRNA IGS.

Source of variation	% Variation^a^	Nm^b^
Among climates	12**	3.77
Among provinces^c^	14*	3.01
Among provinces in humid continental climates^c^	22*	1.74
Among provinces in Mediterranean climate	4 ns	11.23
Among provinces in cold semiarid climates	15 ns	2.83

a Variance among populations is given as a percentage of the total variance.

b Gene flow.

c Without Semnan.

*P < .05; **P < .01; and NS: not significant.

### Analysis of 16S rRNA, protein-encoding housekeeping, and nodulation genes

The *recA, glnII, atpD, nodC*, and 16S rRNA sequences were compared to those of the most closely related bacterial species using the NCBI BLAST program and the EzTaxon Database. Subsequently, the sequences were submitted to the GeneBank/NCBI database through BankIt. Accession numbers for 27 isolates sequenced in this research are available in Table S2 (Supporting Information).

The ANI of the 16S rRNA sequences of selected strains and type strains such as *R. laguerreae* FB206^T^, *R. ruizarguesonis* UPM1133^T^, *R. leguminosarum* USDA 2370^T^, *R. leguminosarum* bv. viciae 248, *R. leguminosarum* GLR17, *R. sophorae* CCBAU 03386^T^, *R. brockwellii* CC275e^T^, *R. johnstonii* 3841^T^, and *R. beringeri* SM51^T^ ranged between 99.5% and 100%. Therefore, it was not feasible to differentiate the species solely by comparing the 16S rRNA gene sequences.

Nucleotide polymorphisms of the 27 representative strains for *recA, glnII, atpD*, and *nodC* were calculated (Table 5). Nucleotide diversities for *recA, glnII, atpD*, and *nodC* were 0.016, 0.034, 0.008, and 0.046, respectively. The number of haplotypes ranged from 10 to 21.

**Table 5.** Descriptive statistics of nucleotide polymorphisms.

Locus	L	h	Hd	S	π
*recA*	478	10	0.89	21	0.016
*glnII*	648	12	0.92	54	0.034
*atpD*	518	11	0.92	15	0.008
*nodC*	884	21	0.97	95	0.046
*recA/glnII/atpD*	1644	22	0.98	90	0.021

L: length of the sequences; h: number of haplotypes, Hd: haplotype (gene) diversity; S: number of polymorphic (segregating) per sites; and π: nucleotide diversity.

**Figure 3.**
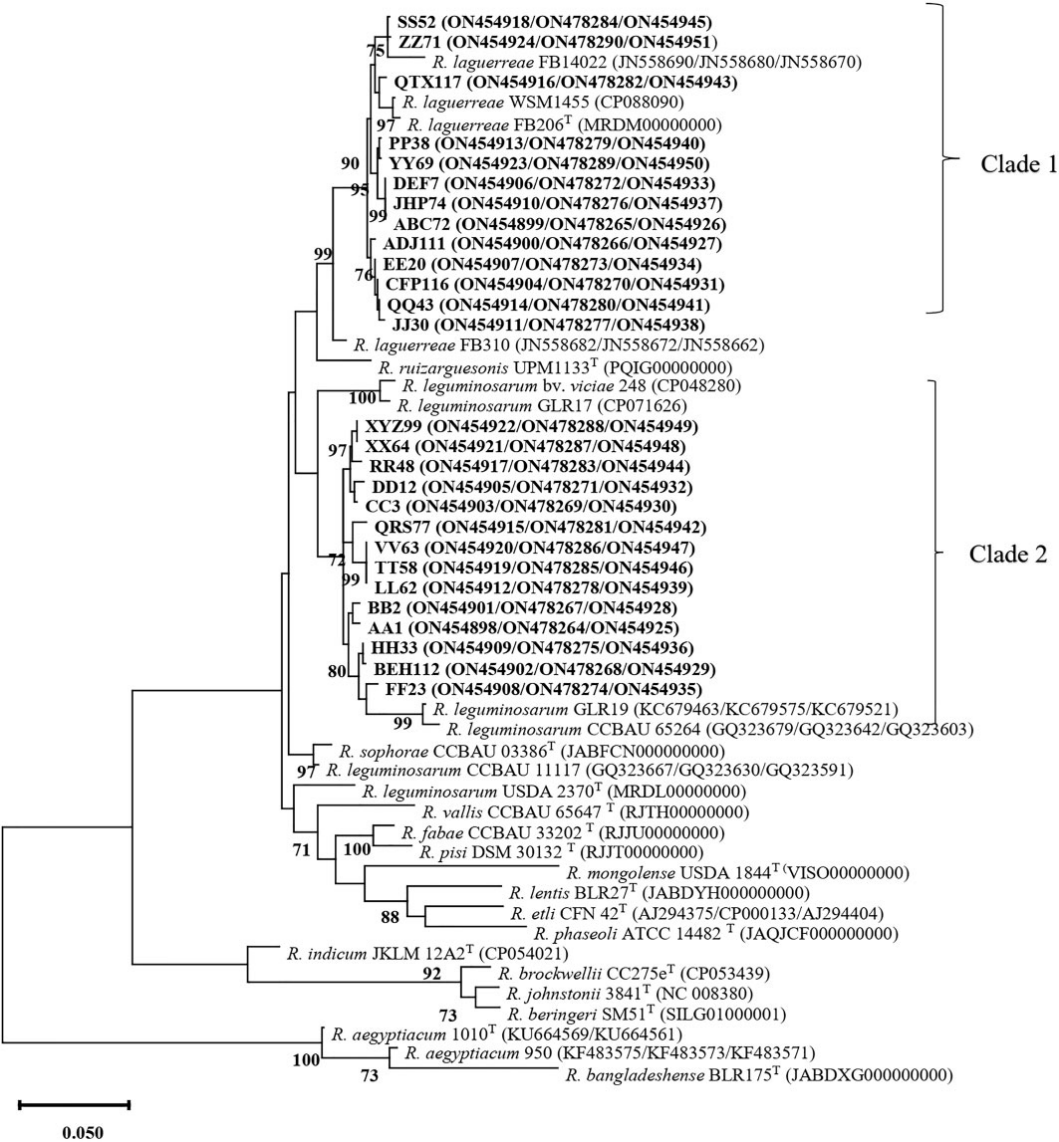
The concatenated dendrogram was constructed based on the *recA, glnII*, and *atpD* gene sequences of the selected rhizobia strains. The bootstrap test was performed with 1000 replications, and values higher than 70% were written on the relevant branches.

**Figure 4.**
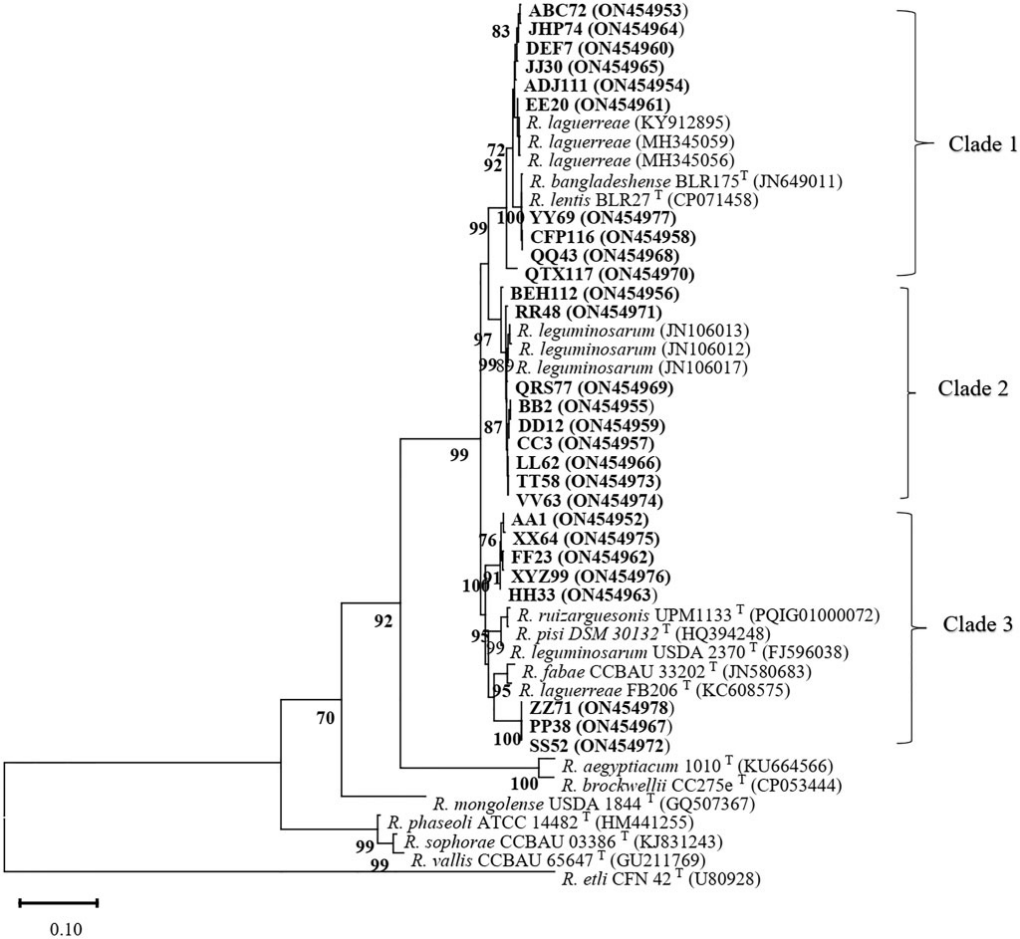
The maximum-likelihood tree based on the *nodC* gene sequences of the selected rhizobia strains. The bootstrap test was performed with 1000 replications, and values higher than 70% were written on the relevant branches.

Aligned sequences from the *recA, glnII*, and *atpD* genes were concatenated, resulting in 1644 bp positions. The phylogenetic analysis began by comparing the concatenated sequences and the nodC gene obtained in this study with those previously published in GenBank. All sequence accession numbers used in phylogenetic analyses are shown in parentheses in Figs 3 and 4.

The concatenated tree based on *recA*–*glnII–a tpD* gene sequences displays two distinct clades (Fig. 3). Clade 1 consists of 13 isolates, which share similarity with strains such as *R. laguerreae* FB206T, *R. laguerreae* FB14022, and *R. laguerreae* FB310 from Tunisia, as well as *R. laguerreae* WSM1455 from Greece. Conversely, Clade 2 encompasses 14 isolates that exhibit similarity to related genes found in *R. leguminosarum* bv. *viciae* 248 from UK, *R. leguminosarum* GLR17 from Germany, *R. leguminosarum* CCBAU 65264 from China, and *R. leguminosarum* GLR19 from Bangladesh.

The ANI values of the concatenated sequences of rhizobial strains in Clade 1 and *R. laguerreae* FB206^T^ ranged from 95.4% to 96%. However, the ANI percentages between strains in clade 2 and *R. laguerreae* FB206^T^ were between 91.3% and 92.6%. Conversely, The ANI values of the concatenated sequences of rhizobial strains in Clade 2 and *R. leguminosarum* bv. *viciae* 248, as well as *R. leguminosarum* GLR17 exceeded 95%. However, the ANI percentages between strains in Clade 1 and these two species were lower than 95%.

Based on the *nodC* gene sequences, the studied strains are categorized in three distinct clades (Fig. 4). Clade 1 comprises 14 isolates, which cluster together with similar strains of *R. leguminosarum* from Syria. Clade 2 consists three isolates with a high Bootstrap value (91%), grouping with similar strains of *R. fabae* CCBAU 33202^T^ and *R. laguerreae* FB206^T^ from China and Tunisia, respectively. Clade 3 encompasses 10 isolates with a robust Bootstrap value (99%) along with similar strains of *R. lentis* BLR27^T^ and *R. bangladeshense* BLR175^T^ from Bangladesh, *R. laguerreae* PEPV11 and *R. laguerreae* MLSC04 from Spain, and *R. laguerreae* LMR614 from Morocco. Based on the *nodC* gene sequence, strains belonging to the same species were not consistently grouped within the same clade. This observation suggests that the *nodC* gene region may not have the capability to effectively differentiate between *R. leguminosarum* and *R. laguerreae*, possibly due to its location on plasmids and susceptibility to horizontal gene transfer.

## Discussion

16S rRNA PCR amplification is one of the best techniques for the taxonomic determination of large databases of bacteria. However, it is inadequate for effective identification and discrimination of *Rhizobium* species. Contrary to the slow rate of evolution of the 16S rRNA gene, which does not allow for proper identification of closely related species, housekeeping genes provide a better resolution to differentiate *Rhizobium* species.

Based on the sequence alignment of the 16S rRNA gene and ANI values, it was observed that the sequences of our strains and the *R. leguminosarum* species complex (Rlc) were highly similar, with ANI values ranging between 99.5% and 100%. Therefor the 16S rRNA gene was not a suitable region for identifying and distinguishing the genospecies. Previous studies have also demonstrated that identical 16S rRNA sequences can be shared by *Rhizobium* species like *R. laguerreae* and *R. leguminosarum* (Taha et al. 2018). Young et al. (2021) further noted that the 16S ribosomal RNA sequence tends to be overly conserved, limiting its discriminatory power among closely related species. Notably, the full-length 16S sequences of all five type strains within the Rlc—*R. leguminosarum, R. laguerreae, R. sophorae, R. ruizarguesonis*, and *R. indicum*—were found to be identical. Even the sequence of *R. anhuiense*, and that of the more distantly related *R. acidisoli*, proved identical. Therefore, *recA, atpD*, and *glnII* genes were utilized for molecular identification of lentil rhizobia.

In this study, 116 isolates were obtained for PCR-RFLP analysis of the 16S–23S rRNA IGS based on the plant infection assay. This technique has been widely utilized to explore the diversity within rhizobia strains. In our study, 116 isolates from nine provinces were classified into 20 groups, leaving seven strains unclustered utilizing BshFI and MspI restriction enzymes. Our study unveiled an intermediate level of chromosomal diversity. Palmer and Young (2000) identified 25 genotypes out of 285 isolates using HaeIII, considering it within the typical variation range for *R. leguminosarum*. They conclude that rhizobial diversity can be influenced by differences between two management regimens in arable and grass sites. Conversely, Depret and Laguerre (2008) detected 28 haplotypes among 1100 isolates from a single site in France using HaeIII. Lu et al. (2009) reported 33 clusters or single strains among 174 rhizobia strains in three ecological regions of China using MspI, HhaI, and HaeIII. In contrast, Mutch et al. (2003) found only nine different types among 625 isolates using TaqI.

Based on the AMOVA approach, our study found significant genetic variation among provinces and four climatic zones in rhizobia strains associated with lentils. This finding is consistent with the observations of Riah et al. (2014), who conducted a similar study using HaeIII (an isoschizomer of BshF I) for IGS RFLP analysis of rhizobia populations associated with lentils and peas in Eastern Algeria. In their study, significant differentiation in the distribution of IGS haplotypes was observed between populations from different ecoclimatic zones, namely subhumid and semiarid zones. Additionally, they found significant differentiation between sites within each ecoclimatic zone. In contrast to the findings of Riah et al. (2014), our study revealed significant differentiation among provinces in the humid continental climate, while differentiation among provinces in the Mediterranean climate and cold semiarid climate was not significant. These variations in findings may reflect differences in the specific environmental conditions, agricultural practices, and genetic diversity of rhizobia populations across different geographic regions and ecoclimatic zones.

The comparison of genetic diversity within and among populations across different climates in our study revealed that the majority (88%) of the diversity exists within populations, while the amount of genetic diversity among populations is relatively low. This low level of genetic diversity among populations can be attributed to the high degree of gene flow, which was calculated to be 3.77. Wright (1978) classified gene flow (Nm) into three categories: high (≥1.0), medium (0.250–0.99), and low (0.0–0.249). According to this classification, gene flow between populations is considered to occur when Nm ≥ 1. In our study, the data indicate a high level of gene flow among the populations. The observed high gene flow among populations could be due to the absence of physical barriers that limit gene flow, facilitating the movement of rhizobia strains across different regions (Muthini et al. 2014). Human activities, such as the transfer of plants, soils, and the circulation of lentil seeds through trading within the region, may have also contributed to the maintenance of genetic diversity among rhizobia populations in Iran. These findings align with those of Elboutahiri et al. (2010), who reported a higher proportion of significant genetic variation distributed within regions (89%) than among regions (11%) in *Sinorhizobium meliloti* and *S. medicae* obtained from drought- and salt-affected regions of Morocco.

In this study, we observed that *R. leguminosarum* and *R. laguerreae* were present in most provinces of Iran, including North Khorasan, Ardabil, Zanjan, Kohgiluyeh-Boyer Ahmad, Lorestan, and Fars, albeit with varying frequencies. For instance, in North Khorasan and Zanjan provinces, 83% and 79% of the isolates, respectively, belonged to *R. leguminosarum*. Conversely, in Ardabil and East Azerbaijan provinces, the dominant species was *R. laguerreae*, with 61% and 100% of the isolates belonging to this species, respectively. In some provinces such as Lorestan, Fars, and Kohgiluyeh-Boyer Ahmad, both species were observed with almost equal frequency. Geographical location appears to play a role in the distribution of these species. For example, North Khorasan province, where most isolates were related to *R. leguminosarum*, is situated in the northeast of Iran. Conversely, *R. laguerreae* was dominant in East Azerbaijan and Ardabil provinces, located in the northwest of Iran. In the western and southwestern parts of Iran, including Lorestan and Kohgiluyeh-Boyer Ahmad provinces, both species were present almost equally. It is noteworthy that in Lorestan and Ardabil provinces, both species were isolated from the same soil, indicating their coexistence within these regions.

Previous studies have revealed variability in the main symbiotic bacteria of *L. culinaris* (lentils) across different regions. In several countries such as West Asia–North Africa, Algeria, Canada, and France, *R. leguminosarum* has been identified as the primary symbiont of lentils (Hynes and O’Connell 1990, Moawad and Beck 1991, Laguerre et al. 1992, Tian et al. 2010). However, research conducted in Morocco by Taha et al. (2018) demonstrated that the dominant species associated with lentils was *R. laguerreae*, indicating regional variation in symbiotic partners. Similarly, investigations in Bangladesh, utilizing multiple genes including 16S rRNA, *glnII, atpD, recA*, and nodulation genes *nodA, nodC*, and *nodD*, revealed genetic similarity with *R. etli* and *R. leguminosarum* species (Rashid et al. 2012). Subsequent studies by Rashid et al. (2015) identified additional symbiotic species including *R. lentis, R. bangladeshense*, and *R. binae* in Bangladesh. Moreover, Dhaoui et al. (2016) reported the presence of various endosymbiotic bacteria of *L. culinaris* in Tunisia, including *R. leguminosarum* symbiovar *trifolii, Ensifer numidicus*, and *Mesorhizobium amorphae* symbiovar *cicero*, highlighting the diversity of symbiotic associations in lentil crops across different geographic regions. These findings underscore the importance of understanding regional variations in symbiotic relationships for effective lentil cultivation and crop management strategies.

In our study, the incongruence observed in the phylogenetic trees constructed from individual protein-coding housekeeping genes (*recA* and *atpD*) and the concatenated sequences of the *recA*–*glnII– atpD* gene sequences underscores the complexity of evolutionary relationships within lentil rhizobia populations. This phenomenon is not uncommon in phylogenetic studies, as different genes may evolve at different rates or be subject to different selective pressures, leading to discordant tree topologies. However, the consistent tree topology observed between the *glnII* gene sequences and concatenated sequences (data not shown). This is in line with previous findings by Ribeiro et al. (2013) and highlights the importance of considering multiple genes for accurate phylogenetic inference. The study by Young et al. (2021) further supports the notion that partial sequences of housekeeping genes may not always reflect the true phylogenetic relationships among strains, especially when alleles within a genospecies do not form distinct clades. Therefore, relying solely on single genes for phylogenetic analysis may lead to incomplete or inaccurate interpretations of evolutionary history. The phylogenetic analysis of the concatenated *recA*–*glnII– atpD* gene sequences revealed distinct clades corresponding to different species. Isolates within clade 1 showed similarity to *R. laguerreae* isolates from Tunisia and Greece, suggesting a close evolutionary relationship. Conversely, isolates belonging to *R. leguminosarum* formed a distinct clade along with isolates from UK and Germany, indicating distinct species boundaries and evolutionary lineages.

The genomic evidence presented by Young et al. (2021) highlights the considerable diversity within the Rlc, indicating that it comprises multiple distinct species. By constructing a phylogeny based on concatenated sequences of 120 universal genes and calculating pairwise ANI between all genomes, they delineated 18 distinct genospecies within the Rlc. Additionally, they identified seven unique strains that do not fit into any of these genospecies. Among the 18 distinct genospecies identified, eight of them include the type strains of named species: *R. laguerreae, R. sophorae, R. ruizarguesonis, R. indicum, R. brockwellii, R. johnstonii, R. beringeri*, and *R. leguminosarum* itself (Young et al. 2023).

The Genome Taxonomy Database employs a 95% ANI threshold to define species and aims to incorporate and classify all available genomes (Parks et al. 2020). In our study, ANI analysis revealed that our strains are related to two species within the *Rhizobium* genus: *R. leguminosarum* and *R. laguerreae*. The ANI values obtained for the concatenated sequences of rhizobial strains in Clade 1 and *R. laguerreae* FB206^T^ ranged from 95.4% to 96%, indicating a close relationship between these strains. Similarly, the ANI values of the concatenated sequences of rhizobial strains in Clade 2 and *R. leguminosarum* bv. *viciae* 248, as well as *R. leguminosarum* GLR17, exceeded 95%, suggesting their affiliation with *R. leguminosarum* species. Comparing ANI values between our strains and three newly introduced species (*R. brockwellii* CC275e ^T^, *R. johnstonii* 3841^T^, and *R. beringeri* SM51^T^) revealed ANI values ranging between 73% and 79%, indicating our strains are distinct from these species. Moreover, the ANI values of our strains with *R. ruizarguesonis* UPM1133^T^, which is closely related to Clade 1 in Fig. 3, ranged between 91% and 93%.

In conclusion, the findings of this study provide valuable insights into the nodulation patterns and genetic diversity of lentil-nodulating rhizobia across various provinces of Iran. It was observed that *L. culinaris* could nodulate with both *R. leguminosarum* and *R. laguerreae* in different climatic zones. Specifically, *R. leguminosarum* appeared to be more compatible in semiarid climates, such as those found in North Khorasan and Ghazvin provinces, while *R. laguerreae* showed a higher compatibility in humid continental climates, as seen in Ardabil and East Azerbaijan provinces. Interestingly, Lorestan and Kohgiluyeh-Boyer Ahmad provinces, characterized by a Mediterranean climate, exhibited the presence of both species with almost equal frequency. This suggests that the climatic conditions in these provinces may support the growth and nodulation of both *R. leguminosarum* and *R. laguerreae*. However, the study also underscores the need for further research to explore the genetic diversity of lentil-nodulating rhizobia across a wider range of climatic conditions. Understanding the genetic diversity and adaptation of rhizobia to different environments is crucial for optimizing lentil production and enhancing symbiotic N-fixation in agricultural systems. In summary, the present study sheds light on the nodulation dynamics of lentil with *R. leguminosarum* and *R. laguerreae* across diverse climatic zones in Iran and highlights avenues for future research to deepen our understanding of rhizobial diversity and adaptation in legume crops.

## Supplementary Material

fiae046_Supplemental_File
