# Supplementary material for: Geographical and climatic distribution of lentil-nodulating rhizobia in Iran
Source: FEMS Microbiol Ecol. 2024 Apr 8;100(5):fiae046. doi: 10.1093/femsec/fiae046 (PMC11044965; doi:10.1093/femsec/fiae046)
Supplement: fiae046_Supplemental_File [file fiae046_supplemental_file.docx]

**Table S1.** Geographical origin and IGS-RFLP groups of rhizobial isolates.

| Province | Sample  type | Number of isolates | Isolates | IGS-RFLP group, respectively |
| --- | --- | --- | --- | --- |
| North Khorasan | Soil | 1 | AA1 | 1 |
|  | Soil | 1 | BB2 | 21 |
|  | Soil | 1 | CC3 | 3 |
|  | Soil | 1 | KH4 | 2 |
|  | Soil | 1 | KH5 | 1 |
|  | Soil | 1 | KH6 | 1 |
|  | Soil | 1 | KH7 | 2 |
|  | Soil | 1 | KH8 | 1 |
|  | Soil | 2 | KH10, KH11 | 17, 17 |
|  | Soil | 2 | DD12, KH13 | 2, 2 |
|  | Soil | 1 | KH14 | 11 |
|  | Soil | 3 | KH15, KH16, KH17 | 2, 3, 1 |
|  | Soil | 2 | KH18, KH123 | 2, 2 |
| Fars | Soil | 1 | EE20 | 17 |
|  | Soil | 1 | F21 | 4 |
| Ardabil | Soil | 1 | AR22 | 8 |
|  | Soil | 1 | FF23 | 22 |
|  | Soil | 1 | AR24 | 1 |
|  | Soil | 4 | AR25, AR26, AR27, AR28 | 1, 3, 4, 4 |
|  | Soil | 1 | AR29 | 5 |
|  | Soil | 3 | JJ30, AR31, AR32 | 4, 4, 4 |
|  | Soil | 4 | HH33, AR34, AR35, AR36 | 23, 4, 1, 1 |
|  | Soil | 1 | AR37 | 4 |
|  | Soil | 2 | PP38, AR39 | 13, 13 |
|  | Soil | 1 | AR40 | 5 |
|  | Soil | 1 | AR41 | 11 |
|  | Soil | 1 | AR42 | 8 |
|  | Soil | 2 | QQ43, AR44 | 24, 8 |
|  | Soil | 2 | AR45, AR46 | 5, 5 |
|  | Soil | 1 | AR47 | 6 |
|  | Soil | 3 | RR48, AR49, AR50 | 8, 11, 13 |
|  | Soil | 1 | AR51 | 4 |
|  | Soil | 1 | SS52 | 11 |
|  | Soil | 1 | AR53 | 8 |
|  | Soil | 1 | AR120 | 15 |
|  | Soil | 2 | AR54, AR55 | 5, 11 |
|  | Soil | 1 | AR56 | 5 |
| Kohgiluyeh-Boyer Ahmad | Soil | 2 | K57, TT58 | 8, 10 |
|  | Soil | 1 | K59 | 5 |
|  | Soil | 2 | K60, K61 | 5, 5 |
|  | Soil | 2 | LL62, VV63 | 25, 9 |
|  | Soil | 2 | XX64, K65 | 15, 6 |
|  | Soil | 3 | K66, K67, K68 | 10, 10, 9 |
|  | Soil | 1 | YY69 | 12 |
|  | Soil | 2 | K70, ZZ71 | 12, 5 |
|  | Soil | 3 | ABC72, DEF73, JHP74 | 26, 19, 18 |
|  | Soil | 1 | K75 | 10 |
| Lorestan | Soil | 1 | L76 | 11 |
|  | Soil | 2 | QRS77, L78 | 27, 18 |
|  | Soil | 3 | L79, L80, L81 | 19, 12, 1 |
|  | Soil | 1 | L82 | 11 |
|  | Soil | 3 | L83, L84, L85 | 11, 18, 6 |
|  | Soil | 1 | L87 | 11 |
|  | Soil | 2 | L88, L89 | 12, 6 |
|  | Soil | 3 | L90, L91, L92 | 10, 10, 10 |
| Ghazvin | Soil | 1 | GH93 | 3 |
|  | Soil | 1 | GH94 | 3 |
| Zanjan | Nodule | 1 | Z97 | 16 |
|  | Nodule | 1 | Z98 | 3 |
|  | Nodule | 1 | XYZ99 | 7 |
|  | Nodule | 1 | Z100 | 7 |
|  | Nodule | 1 | Z101 | 8 |
|  | Nodule | 1 | Z102 | 2 |
|  | Nodule | 1 | Z103 | 3 |
|  | Nodule | 1 | Z104 | 15 |
|  | Nodule | 1 | Z105 | 3 |
|  | Nodule | 1 | Z106 | 7 |
|  | Nodule | 1 | Z107 | 15 |
|  | Nodule | 1 | Z108 | 5 |
|  | Nodule | 1 | Z109 | 1 |
|  | Nodule | 1 | Z110 | 13 |
| Semnan | Nodule | 1 | ADJ111 | 20 |
| Fars | Nodule | 1 | BEH112 | 6 |
|  | Nodule | 1 | F113 | 20 |
|  | Nodule | 1 | F114 | 8 |
|  | Nodule | 1 | F115 | 4 |
| East Azerbaijan | Nodule | 1 | CFP116 | 16 |
|  | Nodule | 1 | QTX117 | 14 |
|  | Nodule | 1 | AZ118 | 14 |
|  | Nodule | 1 | AZ119 | 16 |

**Table S2.** Identification of bacterial strains based on the *recA*, *glnII*, *atpD*, *nodC*, and 16S rRNA sequences.

| **Accession no.** | | | | | **Bacterial name**  **based on MLSA** | **Isolates** |
| --- | --- | --- | --- | --- | --- | --- |
| ***recA*** | ***glnII*** | ***atpD*** | ***nodC*** | **16S rRNA** |  |  |
| ON454898 | ON478264 | ON454925 | ON454952 | ON428638 | *Rhizobium leguminosarum* | AA1 |
| ON454899 | ON478265 | ON454926 | ON454953 | ON428639 | *Rhizobium laguerreae* | ABC72 |
| ON454900 | ON478266 | ON454927 | ON454954 | ON428640 | *Rhizobium laguerreae* | ADJ111 |
| ON454901 | ON478267 | ON454928 | ON454955 | ON428641 | *Rhizobium leguminosarum* | BB2 |
| ON454902 | ON478268 | ON454929 | ON454956 | ON428642 | *Rhizobium leguminosarum* | BEH112 |
| ON454903 | ON478269 | ON454930 | ON454957 | ON428643 | *Rhizobium leguminosarum* | CC3 |
| ON454904 | ON478270 | ON454931 | ON454958 | ON428644 | *Rhizobium laguerreae* | CFP116 |
| ON454905 | ON478271 | ON454932 | ON454959 | ON428645 | *Rhizobium leguminosarum* | DD12 |
| ON454906 | ON478272 | ON454933 | ON454960 | ON428646 | *Rhizobium laguerreae* | DEF73 |
| ON454907 | ON478273 | ON454934 | ON454961 | ON428647 | *Rhizobium laguerreae* | EE20 |
| ON454908 | ON478274 | ON454935 | ON454962 | ON428648 | *Rhizobium leguminosarum* | FF23 |
| ON454909 | ON478275 | ON454936 | ON454963 | ON428649 | *Rhizobium leguminosarum* | HH33 |
| ON454910 | ON478276 | ON454937 | ON454964 | ON428650 | *Rhizobium laguerreae* | JHP74 |
| ON454911 | ON478277 | ON454938 | ON454965 | ON428651 | *Rhizobium laguerreae* | JJ30 |
| ON454912 | ON478278 | ON454939 | ON454966 | ON428652 | *Rhizobium leguminosarum* | LL62 |
| ON454913 | ON478279 | ON454940 | ON454967 | ON428653 | *Rhizobium laguerreae* | PP38 |
| ON454914 | ON478280 | ON454941 | ON454968 | ON428654 | *Rhizobium laguerreae* | QQ43 |
| ON454915 | ON478281 | ON454942 | ON454969 | ON428655 | *Rhizobium leguminosarum* | QRS77 |
| ON454916 | ON478282 | ON454943 | ON454970 | ON428656 | *Rhizobium laguerreae* | QTX117 |
| ON454917 | ON478283 | ON454944 | ON454971 | ON428657 | *Rhizobium leguminosarum* | RR48 |
| ON454918 | ON478284 | ON454945 | ON454972 | ON428658 | *Rhizobium laguerreae* | SS52 |
| ON454919 | ON478285 | ON454946 | ON454973 | ON428659 | *Rhizobium leguminosarum* | TT58 |
| ON454920 | ON478286 | ON454947 | ON454974 | ON428660 | *Rhizobium leguminosarum* | VV63 |
| ON454921 | ON478287 | ON454948 | ON454975 | ON428661 | *Rhizobium leguminosarum* | XX64 |
| ON454922 | ON478288 | ON454949 | ON454976 | ON428662 | *Rhizobium leguminosarum* | XYZ99 |
| ON454923 | ON478289 | ON454950 | ON454977 | ON428663 | *Rhizobium laguerreae* | YY69 |
| ON454924 | ON478290 | ON454951 | ON454978 | ON428664 | *Rhizobium laguerreae* | ZZ71 |
